# Prediction of the axillary lymph-node metastatic burden of breast cancer by ^18^F-FDG PET/CT-based radiomics

**DOI:** 10.1186/s12885-024-12476-3

**Published:** 2024-06-07

**Authors:** Yan Li, Dong Han, Cong Shen

**Affiliations:** PET/CT Center, The First Affiliated Hospital of Xi’an Jiaotong University, 277 Yanta West Road, Xi’an Shaanxi, Shaanxi 710061 China

**Keywords:** Breast cancer, Axillary lymph nodes, Radiomics, PET/CT

## Abstract

**Background:**

The axillary lymph-node metastatic burden is closely associated with treatment decisions and prognosis in breast cancer patients. This study aimed to explore the value of ^18^F-fluorodeoxyglucose (^18^F-FDG) positron emission tomography (PET)/computed tomography (CT)–based radiomics in combination with ultrasound and clinical pathological features for predicting axillary lymph-node metastatic burden in breast cancer.

**Methods:**

A retrospective analysis was conducted and involved 124 patients with pathologically confirmed early-stage breast cancer who had undergone ^18^F-FDG PET/CT examination. The ultrasound, PET/CT, and clinical pathological features of all patients were analysed, and radiomic features from PET images were extracted to establish a multi-parameter predictive model.

**Results:**

The ultrasound lymph-node positivity rate and PET lymph-node positivity rate in the high nodal burden group were significantly higher than those in the low nodal burden group (*χ*^2^ = 19.867, *p* < 0.001; *χ*^2^ = 33.025, *p* < 0.001). There was a statistically significant difference in the PET-based radiomics score (RS) for predicting axillary lymph-node burden between the high and low lymph-node burden groups. (-1.04 ± 0.41 vs. -1.47 ± 0.41, t = -4.775, *p* < 0.001). The ultrasound lymph-node positivity (US_LNM) (odds ratio [OR] = 3.264, 95% confidence interval [CI] = 1.022–10.423), PET lymph-node positivity (PET_LNM) (OR = 14.242, 95% CI = 2.960–68.524), and RS (OR = 5.244, 95% CI = 3.16–20.896) are all independent factors associated with high lymph-node burden (*p* < 0.05). The area under the curve (AUC) of the multi-parameter (MultiP) model was 0.895, which was superior to those of US_LNM, PET_LNM, and RS models (AUC = 0.703, 0.814, 0.773, respectively), with statistically significant differences (Z = 2.888, 3.208, 3.804, respectively; *p* = 0.004, 0.002, < 0.001, respectively). Decision curve analysis indicated that the MultiP model provided a higher net benefit for all patients.

**Conclusion:**

A MultiP model based on PET-based radiomics was able to effectively predict axillary lymph-node metastatic burden in breast cancer.

**Trial registration:**

This study was registered with ClinicalTrials.gov (registration number: NCT05826197) on May 7, 2023.

## Background

Breast cancer is the most diagnosed cancer and a leading cause of cancer death among women worldwide [1]. Approximately 30–40% of breast cancer patients have axillary lymph-node metastasis, which is closely related to treatment decisions and prognosis [2, 3]. The results of the American College of Surgeons Oncology Group (ACOSOG) Z0011 trial has indicated that axillary lymph-node dissection (ALND) is necessary only when there are three or more positive axillary lymph nodes [4]. Recently, the evaluation of axillary lymph-node metastasis status has shifted from predicting lymph-node metastasis to predicting lymph-node metastatic burden [5]. Patients with low lymph-node metastatic burden can undergo treatment such as total mastectomy or breast-conserving therapy with whole breast radiation, avoiding ALND, without compromising overall survival rates [4, 5]. Patients with high lymph-node metastatic burden benefit from axillary surgery or neoadjuvant chemotherapy, avoiding sentinel lymph-node biopsy [4]. Therefore, the accurate preoperative assessment of axillary lymph-node metastatic burden contributes to the selection of treatment.

Novel nuclides can also be used for breast cancer. For instance, fibroblast activation protein inhibitor (FAPI) specifically binds to fibroblast activation protein and demonstrates high uptake in over 20 types of tumours, including breast cancer. In the diagnosis of axillary lymph-node metastasis in breast cancer, FAPI positron emission tomography (PET)/computed tomography (CT) has exhibited high accuracy and safety [6]. Fluoroestradiol (FES), which is an oestrogen receptor (ER) imaging agent, allows FES PET/CT to be utilised for the diagnosis, staging, and assessment of endocrine therapy effectiveness in ER-positive breast cancer [7]. By using combined fluorodeoxyglucose (FDG) and FES PET/CT imaging, occult lymph-node metastases that are negative for glucose metabolism can be detected. However, FAPI and FES are not available in all hospitals, and the diagnosis of axillary lymph nodes in breast cancer primarily relies on ^18^F-FDG PET/CT in clinical practice.

Radiomics involves the high-throughput mining of quantitative image features from standard medical images and has become a rapidly advancing field of research in breast cancer [8]. Many radiomics studies are based on breast ultrasound (US) and magnetic resonance imaging (MRI), and some radiomics studies are based on ^18^F-FDG PET/CT for breast cancer diagnosis and staging [9]. However, there are relatively few radiomics studies of axillary lymph-node metastatic load in breast cancer.

Therefore, this study categorised patients into low and high lymph-node burden groups based on a threshold of three lymph-node metastases. The radiomics research complied with the European Association of Nuclear Medicine (EANM)/ Society of Nuclear Medicine and Molecular Imaging (SNMMI) joint guidelines for nuclear medicine radiomics [10]. We investigated the feasibility of predicting axillary lymph-node metastatic burden in breast cancer using ^18^F-FDG PET/CT-based radiomics in combination with US and clinical pathological features.

## Methods

### Clinical data

**Fig. 1:**
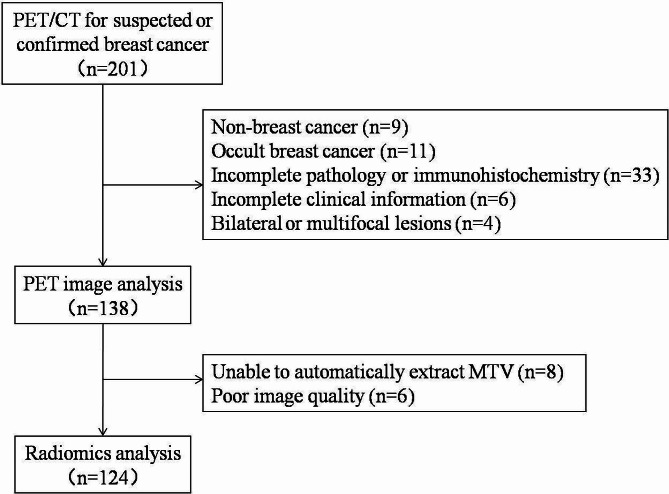
Patient enrolment flow

Patients who underwent PET/CT examinations for breast nodules at the First Affiliated Hospital of Xi’an Jiaotong University between November 2016 and April 2022 were retrospectively collected through the Picture Archiving and Communication System and Hospital Information System systems. The patient enrolment process is depicted in Fig. 1. This study aligned with the principles of the Helsinki Declaration and was approved by the ethics committee of our hospital. All data were anonymized prior to analysis. Tumour staging was based on the Eighth Edition American Joint Committee on Cancer staging manual [11]. The current study was approved by the Ethics Committee of the First Affiliated Hospital of Xi’an Jiaotong University (Approval No: IRB-SOP-AF-16), funded by the Department of Science and Technology of Shaanxi Province (grant no. 2023-YBSF-480), and registered with ClinicalTrials.gov (registration no. NCT05826197).

The inclusion criteria were as follows: patients who had undergone ^18^F-FDG PET/CT examination for breast nodules; adult female patients who were pathologically diagnosed with breast cancer (age ≥ 18 years); no surgery, radiotherapy, or chemotherapy prior to the ^18^F-FDG PET/CT examination; an interval between ^18^F-FDG PET/CT examination and biopsy/surgery ≤ 2 weeks; and complete clinical and pathological data.

The exclusion criteria were as follows: incomplete or poor-quality images, multifocal or bilateral lesions, lesions with no increased FDG uptake, metabolic tumour volume (MTV) could not be segmented automatically, and concurrent presence of other malignancies.

### PET/CT imaging methodology

All patients underwent PET/CT examinations using the Philips TF64 PET/CT scanner. The ^18^F-FDG was synthesised using a GE MINItrace cyclotron and Tracerlab FX-FDG synthesiser, with precursor reagents purchased from ABX, Germany. The synthesised ^18^F-FDG had a radiochemical purity of ≥ 95%, met quality control standards, and was suitable for human injection. The patients fasted for at least 6 h, with a fasting blood glucose level of ≤ 12 mmol/L. The ^18^F-FDG was injected into the vein of the contralateral upper limb of the affected breast at a dose of 370 MBq/kg body weight. Patients were encouraged to drink water and remained at rest for 60 min. The scanning range was from the skull vertex to the mid-thigh. The CT scan parameters were as follows: tube voltage of 120 kV, tube current of 300 mA, slice thickness of 5 mm, interslice gap of 5 mm, and 512 × 512 matrix. PET data were acquired in 3D mode for 1.5 min per bed position covering six to seven bed positions. The PET images underwent attenuation correction by using co-registered CT data and were reconstructed using iterative reconstruction and time-of-flight techniques. The image data were then transferred to the Philips Extended Brilliance Workspace (EBW) workstation for post-processing.

### Image assessment

**Fig. 2:**
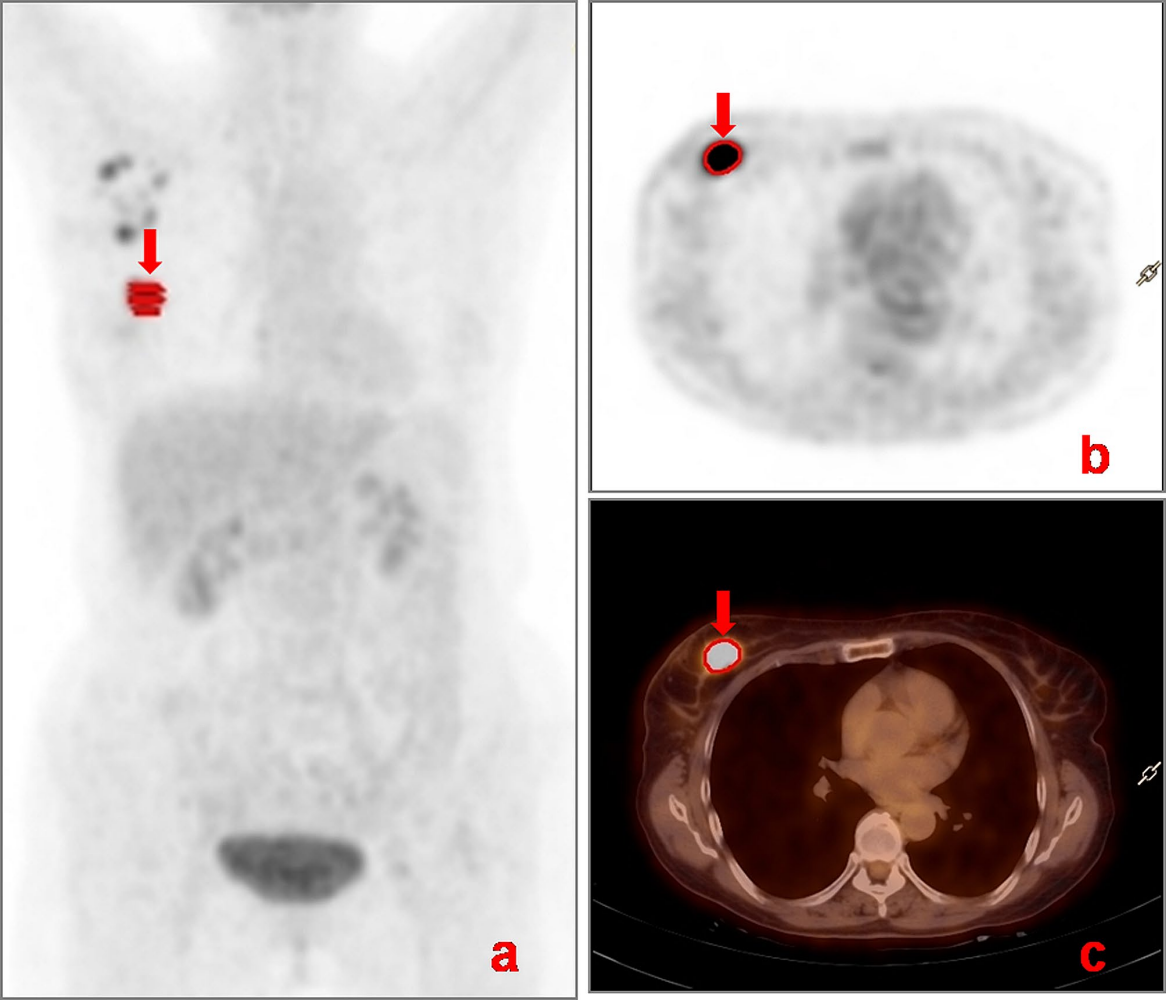
Measurement of PET metabolic parameters by automatic lesion delineation using 3D ROI. A 49-year-old female with right breast cancer. (**a**) Maximum intensity projection image displays the volume of the right breast cancer lesion (arrow) delineated by the red 3D ROI. (**b**) PET image displays the ROI of the lesion (arrow) in the axial plane. (**c**) Fused image displays the ROI of the lesion (arrow)

The PET/CT images were jointly reviewed by one chief radiologist and one senior attending radiologist from the PET/CT centre. In cases of discrepancies, a consensus was reached through consultation. The lesions were visually assessed, and a 40% threshold was used to automatically delineate the lesions in a 3D region of interest (ROI) for measuring PET metabolic parameters, as shown in Fig. 2, including the mean standard uptake value (SUV_mean_), maximum standard uptake value (SUV_max_), standard deviation of the standard uptake value (SUV_StdDev_), and MTV. The criteria for assessment were as follows: an area of radioisotope uptake greater than that in the surrounding breast tissue was indicative of a breast cancer lesion, whereas a lymph node of radioisotope uptake greater than that in the adjacent muscle tissue indicated a metastatic lymph node.

### Radiomics

**Fig. 3:**
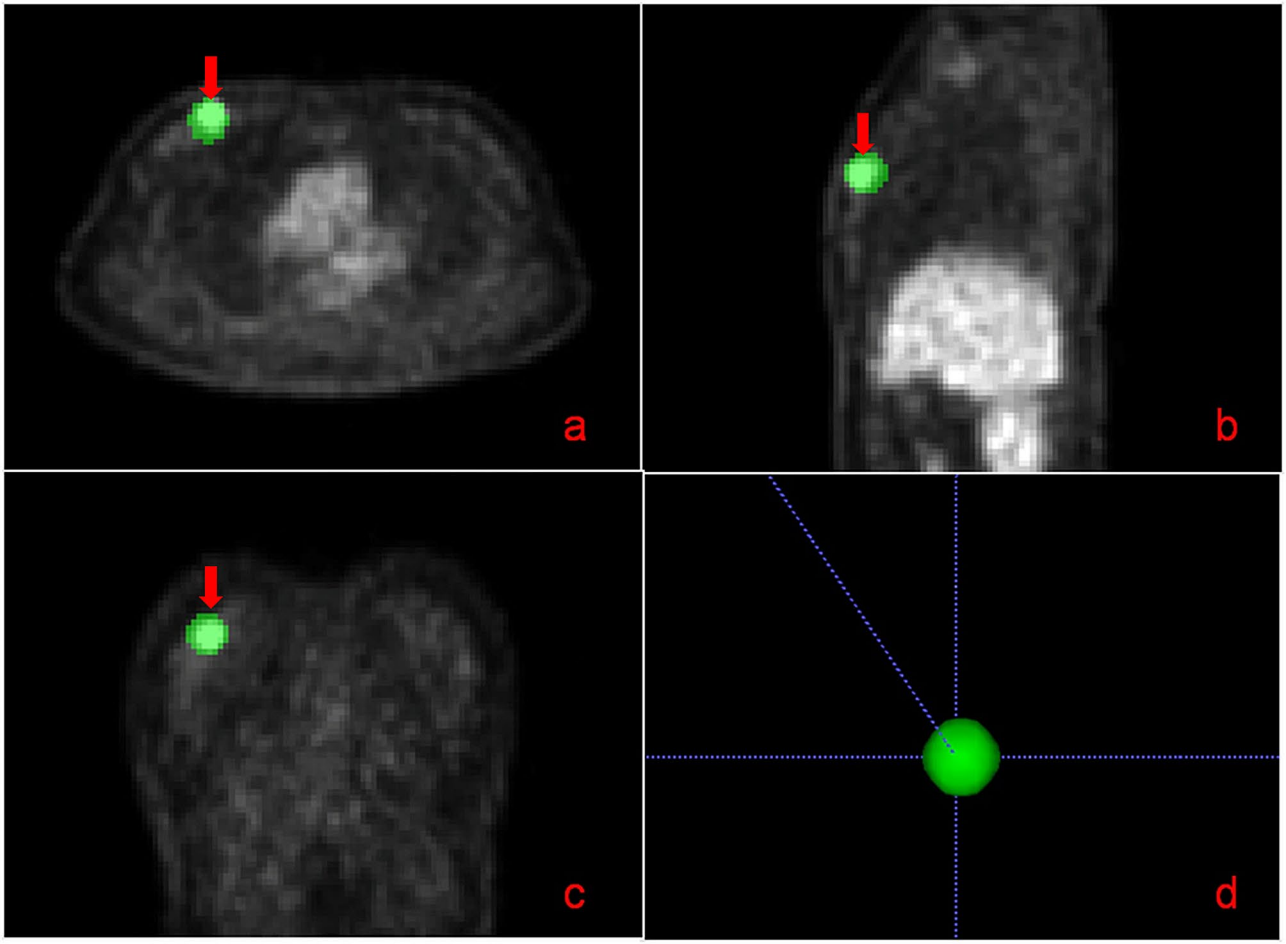
Three-dimensional segmentation of the breast cancer lesion. By using the ITK-SNAP software, the right breast cancer lesion (arrow) was pseudo-coloured with green in the axial (**a**), sagittal (**b**), and coronal (**c**) views, and the tumour lesion segmentation was performed automatically (**d**)

Data acquisition: Raw DICOM data were exported from the EBW workstation. Image segmentation: Image segmentation was performed using ITK-SNAP software [12] (Version 3.6.0, https://itk.org/), with a circular brush style, a brush size of 10, and brush options of 3D. The entire tumour volume on the PET image was delineated as an ROI for segmentation, as shown in Fig. 3. The lesions were marked by the attending radiologist and then verified by the chief radiologist.

An open-source Python package (PyRadiomics 3.0.1 [13]) was applied to extract radiomic features of the ROI, thus resulting in a total of 851 radiomic features being computed. The feature extraction and definition adhere to the image biomarker standardisation initiative. PET radiomics studies were conducted according to the EANM/SNMMI guidelines [10].

### Clinical and pathological characteristics

All breast nodules were classified using the Breast Imaging Reporting and Data System. Breast cancer histological grading was performed using the internationally recognised Nottingham Histologic Grading system. The breast cancer specimens were fixed in 4% formaldehyde solution and embedded in paraffin. They were then cut into 4 μm sections and underwent routine haematoxylin and eosin staining to detect the pathological type and histological grade. Additionally, immunohistochemical staining for ER, progesterone receptor (PR), and human epidermal growth factor receptor 2 (HER2) were performed, as well as Ki67 proliferation index testing. ER positive: ≥10% of tumour cell nuclei stained positive; PR positive: ≥10% of tumour cell nuclei stained positive; HER2 positive: immunohistochemistry score was ≥ 2+; and high Ki67 expression: expression index ≥ 14%.

According to the 2023 National Comprehensive Cancer Network guidelines [14], breast cancer can be classified into luminal A, luminal B, HER2-positive, and triple-negative subtypes. The luminal A subtype is ER positive and/or PR positive, has high PR expression (≥ 20%), is HER2 (-), and has low Ki67 expression (< 14%). The luminal B subtype has two variants: (a) the HER2-negative type is ER positive, is HER2 negative, has high Ki67 expression, and has PR negative or low expression (< 20%), and (b) the HER2-positive type is ER positive, is HER2 positive, has any Ki67 expression, and has any PR status. The HER2-positive subtype is ER negative and/or PR negative and is HER2 positive. The triple-negative subtype is ER negative, PR negative, and HER2 negative.

The grouping of axillary lymph-node metastatic burden was based on the results of the ACOSOG Z0011 trial [4]: three or more lymph-node metastases were classified as the high nodal burden (HNB) group, whereas less than three lymph-node metastases were classified as the low nodal burden (LNB) group.

### Statistical analysis

Statistical analysis was performed using R v.4.1.0 and SPSS v.27.0 (IBM Corp., New York), with a significance level set at α = 0.05. Continuous variables were expressed as mean ± standard deviation. Independent samples t-test was used for comparing continuous data between two groups that were normally distributed and had homogeneity of variance; otherwise, the Mann–Whitney U test was used. Count variables were expressed as frequencies, and the comparison between the two groups was performed using the χ^2^ test. The dimensionality reduction of radiomic features was achieved using the least absolute shrinkage and selection operator (LASSO). The radiomics score (RS) was calculated based on the reduced features and the linear weighting of their coefficients. Variables with significant differences between the two groups were included in a multivariate binary logistic regression analysis to develop a multivariate prediction model and plot the nomogram. The discrimination of the nomogram was assessed using receiver operating curve (ROC) curve analysis. The cut-off value of the ROC curve was calculated based on the maximum Youden index, and sensitivity and specificity were also calculated. The DeLong test was used to compare the area under the curve (AUC) between the parameters. Further internal validation of the multi-parameter (MultiP) model was performed using 1000 bootstrap resamples, and the adjusted AUC was calculated. Simultaneously, a calibration curve was plotted to assess the calibration of the MultiP model. A decision curve was further plotted to evaluate the net benefit of the MultiP model across all patients. Differences with *p* < 0.05 were considered statistically significant.

## Results

### Comparison of general information

A total of 124 patients were included, with ages ranging from 20 to 76 years and a mean age of 49 years. Clinical and pathological characteristics were compared between the LNB group (*n* = 98) and the HNB group (*n* = 26) to identify potential diagnostic biomarkers for axillary lymph-node metastatic burden. The proportion of positive US_LNM was higher in the HNB group than in the LNB group, with a statistically significant difference (*χ*^2^ = 19.867, *p* < 0.001). The proportion of positive PET lymph-node positivity (PET_LNM) was also higher in the HNB group than in the LNB group, with a significant difference (*χ*^2^ = 33.025, *p* < 0.001; Table 1). Meanwhile, there were no significant differences between the LNB and HNB groups in terms of age, tumour location, quadrant distribution, Breast Imaging Reporting and Data System classification, T stage, molecular subtype, pathological type, grade, SUV_max_, SUV_mean_, SD, and MTV (*p* > 0.05; Table 1).

**Table 1:** Comparison of general information of patients in two groups

Parameters	LNB (≤ 2) (*n* = 98)	HNB (≥ 3) (*n* = 26)	t/χ^2^	*p*
**Age**	48.57 ± 11.66	51.12 ± 11.11	-0.999	0.320
**US_BIRADS**			7.543	0.110
Bi-rads 1	1	1		
Bi-rads 3	2	1		
Bi-rads 4	58	8		
Bi-rads 5	20	10		
Bi-rads 6	17	6		
**Tumour location**			0.066	0.798
Left	50	14		
Right	48	12		
**Quadrant distribution**			10.056	0.074
Outer upper	38	10		
Outer lower	12	3		
Inner upper	31	5		
Inner lower	7	0		
Middle upper	3	4		
Middle lower	7	4		
**US_LNM**			19.867	< 0.001
Negative	85	12		
Positive	13	14		
**T stage**			6.856	0.144
Tis	3	0		
T1	54	8		
T2	32	13		
T3	6	3		
T4	3	2		
**PET_LNM**			33.025	< 0.001
Negative	69	2		
Positive	29	24		
**Subtypes**			1.938	0.380
Invasive ductal carcinoma	85	25		
Invasive lobular carcinoma	10	1		
Ductal carcinoma in situ	3	0		
**Grade**			2.865	0.239
G1	3	0		
G2	44	8		
G3	51	18		
**Mol-subtypes**			3.304	0.347
Luminal A	25	4		
Luminal B	31	13		
HER2-positive	26	5		
Triple-negative	16	4		
**SUVmax**	8.04 ± 4.36	8.67 ± 6.11	-0.601	0.549
**SUVmean**	4.31 ± 2.35	4.03 ± 2.55	0.524	0.601
**SD**	1.28 ± 0.85	1.41 ± 1.45	-0.587	0.558
**MTV**	33825.73 ± 152299.83	28968.19 ± 30363.31	0.161	0.872

### LASSO regression and calculation of RS

**Fig. 4:**
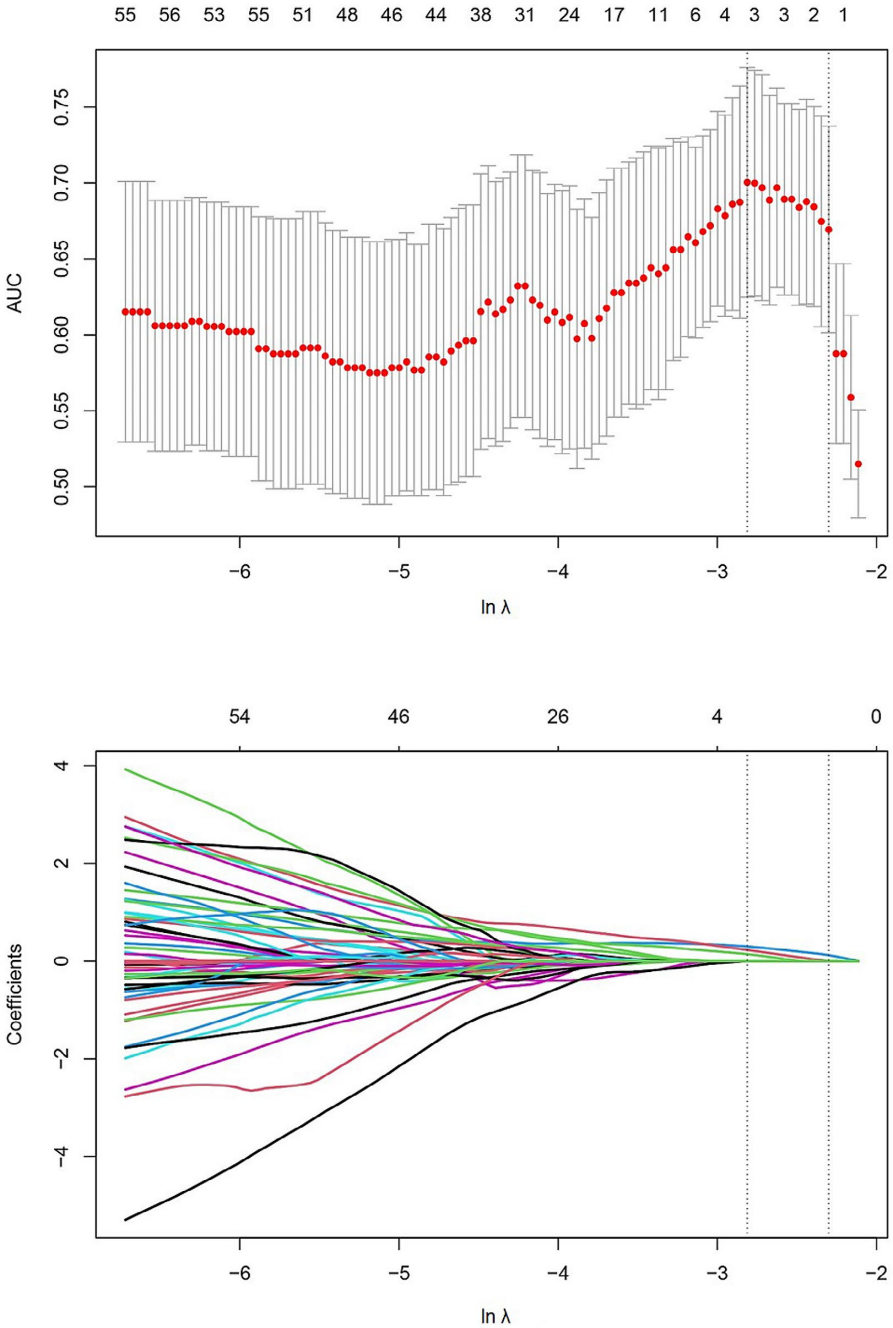
Cross-validation plot with LASSO regression and coefficient plot, the optimal lnλ of -2.812 was obtained through cross-validation. The upper horizontal axis represents the number of radiomic features corresponding to the model. In Fig. 4A, the two vertical dashed lines represent the two logarithmic values (λ) of the minimum mean square error and the minimum mean square error plus 1 standard deviation obtained through cross-validation. In Fig. 4B, as the logarithm (λ) increases, the coefficients of the radiomic features gradually shrink towards 0, and the number of features reduces from the logarithm (λ) of the minimum mean square error to 4

The radiomic features were standardised using Z-score normalisation, followed by dimensionality reduction using LASSO regression, and the optimal lnλ of -2.812 was determined through cross-validation, as shown in Fig. 4.

The RS for axillary lymph-node metastatic burden was calculated based on the three reduced radiomic features and the linear weighting of their coefficients. .

**Fig. 5:**
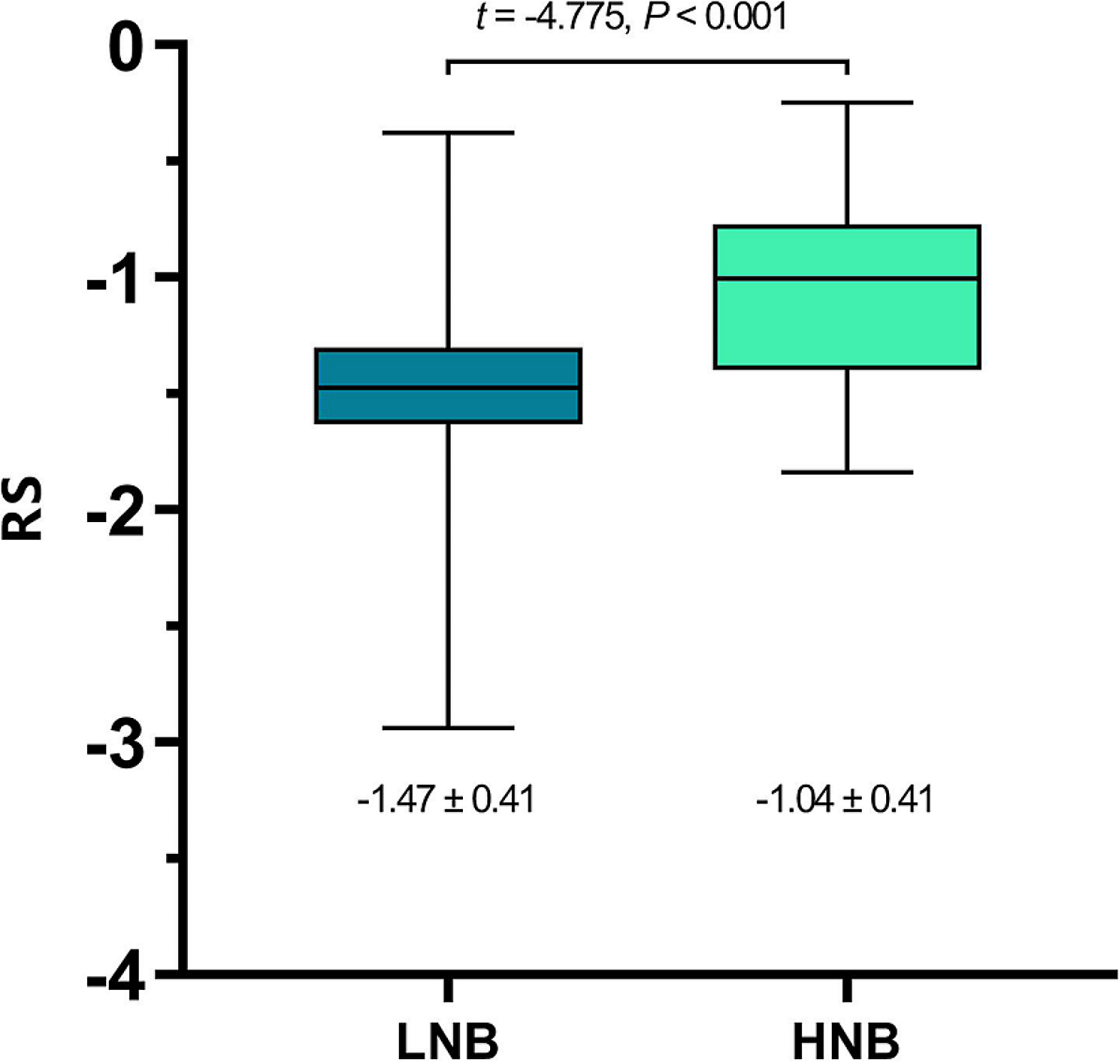
Box plot comparing the RS of the two groups

The RS for LNB group was − 1.47 ± 0.41, whereas the RS for the HNB group was − 1.04 ± 0.41. The difference between the two was statistically significant (*t* = -4.775, *p* < 0.001), as shown in Fig. 5.

### Multivariate logistic regression analysis

**Fig. 6:**
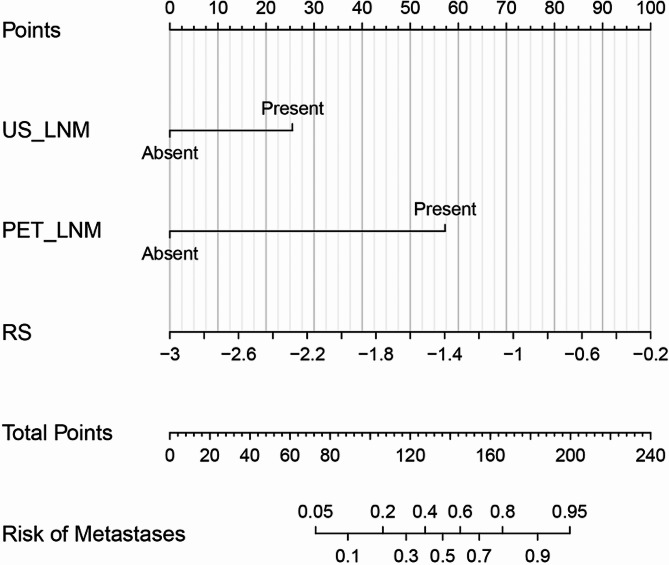
Nomogram of axillary lymph-node metastatic burden parameters based on the results of multivariable logistic regression analysis

The US_LNM, PET_LNM, and RS were included in a multivariate logistic regression analysis, and the results showed that US_LNM (odds ratio [OR] = 3.264, 95% confidence interval [CI] = 1.022–10.423), PET_LNM (OR = 14.242, 95% CI: 2.960–68.524), and RS (OR = 5.244, 95% CI: 1.316–20.896) were all independent influencing factors for high lymph-node burden (*p* < 0.05), as shown in Table 2. A nomogram was plotted based on the results of the multivariable logistic regression analysis, as shown in Fig. 6.

**Table 2:** Multivariate logistic regression analysis results

Parameters	OR	95% CI	*p*
US_LNM	3.264	1.022–10.423	0.046
PET_LNM	14.242	2.960–68.524	< 0.001
RS	5.244	1.316–20.896	0.019

### Assessment of nomogram

**Fig. 7:**
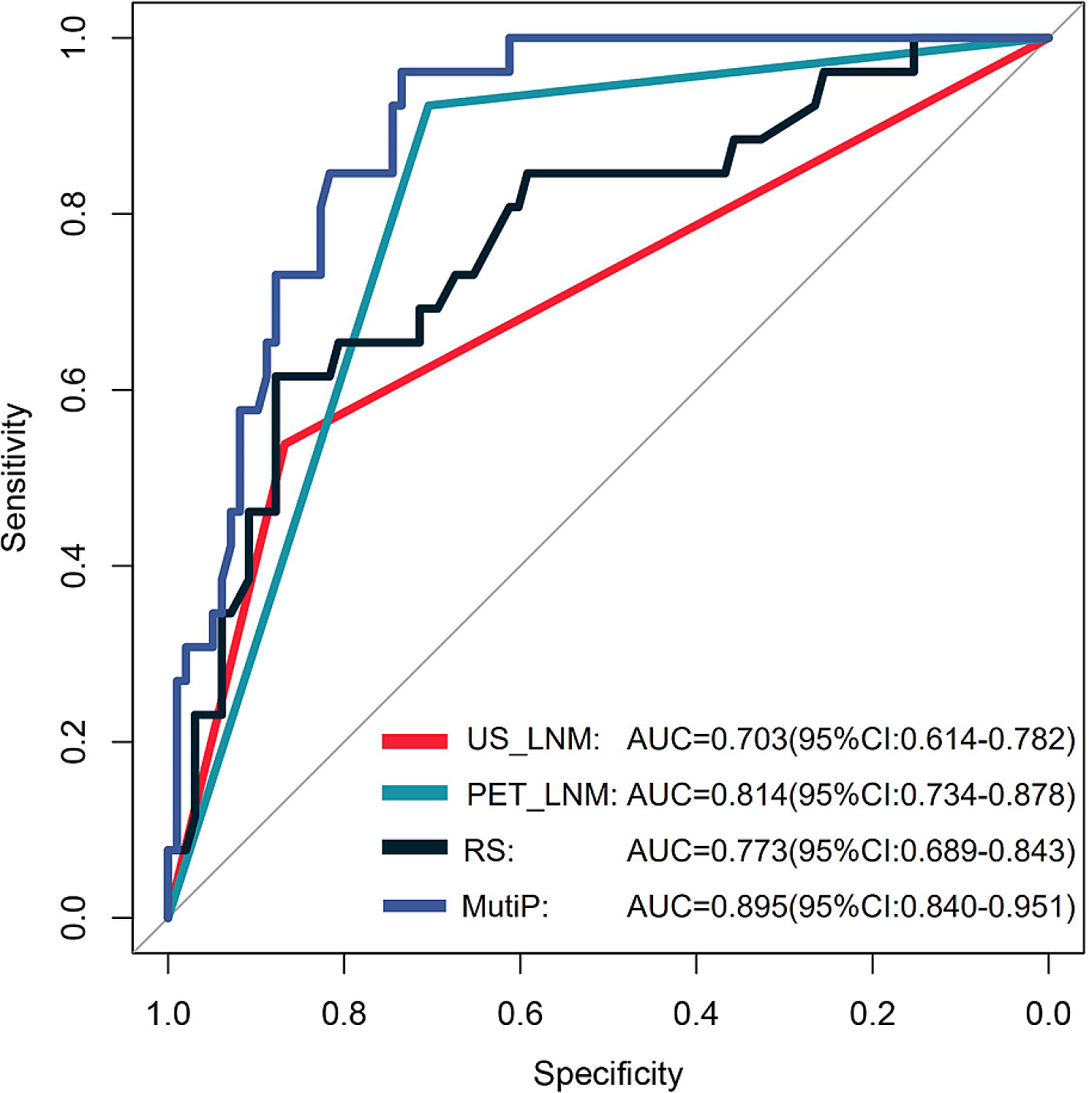
ROC curves of the single- and MultiP nomograms. The AUC of the MultiP model was 0.895, superior to those of US_LNM, PET_LNM, and RS models (AUC = 0.703, 0.814, 0.773, respectively)

**Fig. 8:**
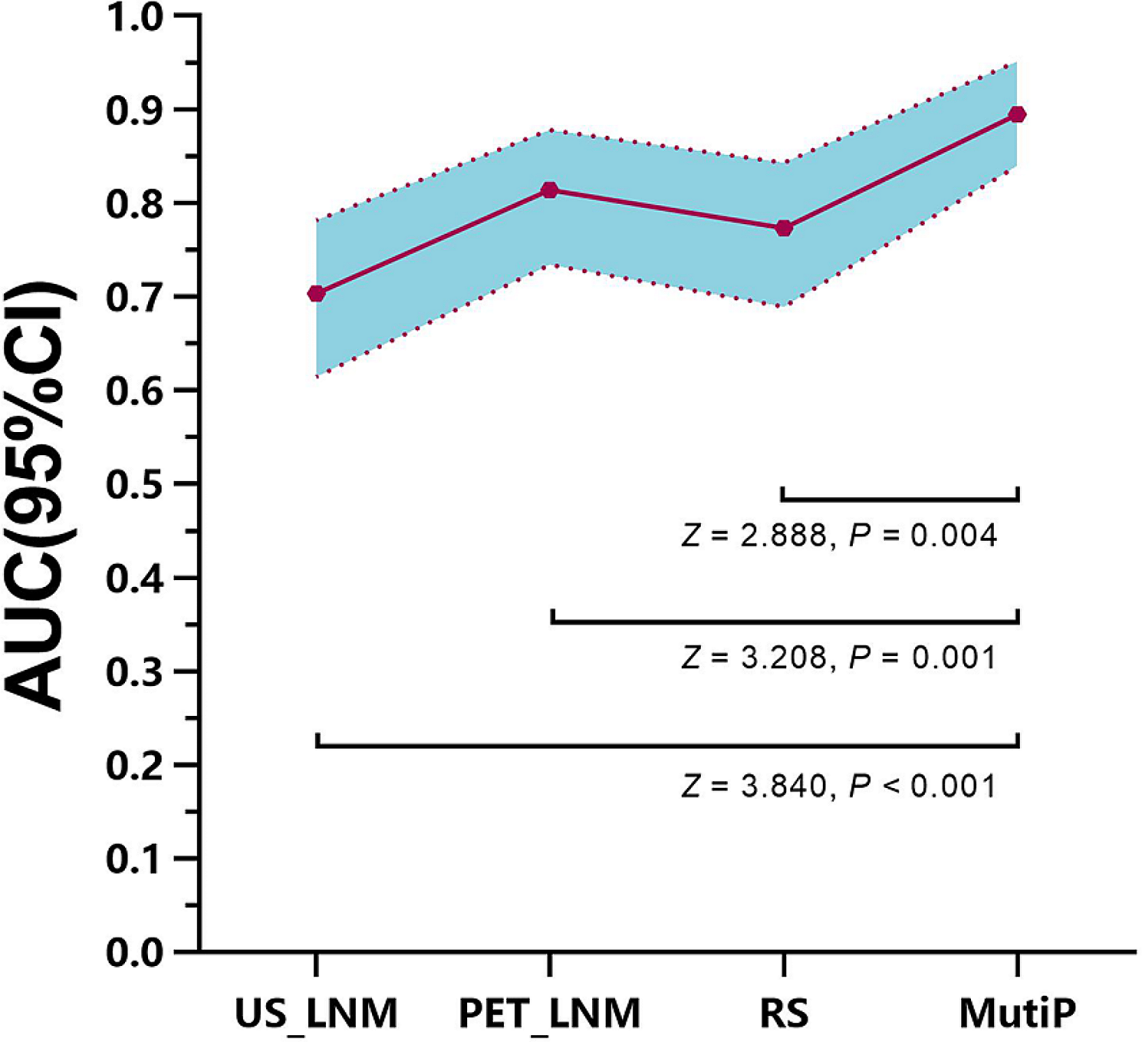
Comparison of ROC curves of the single- and MultiP nomograms. The AUC of MultiP model was compared with those of the US_LNM, PET_LNM, and RS models, and the differences were all statistically significant (Z = 2.888, 3.208, 3.804, respectively; *p* = 0.004, 0.002, < 0.001, respectively)

**Fig. 9:**
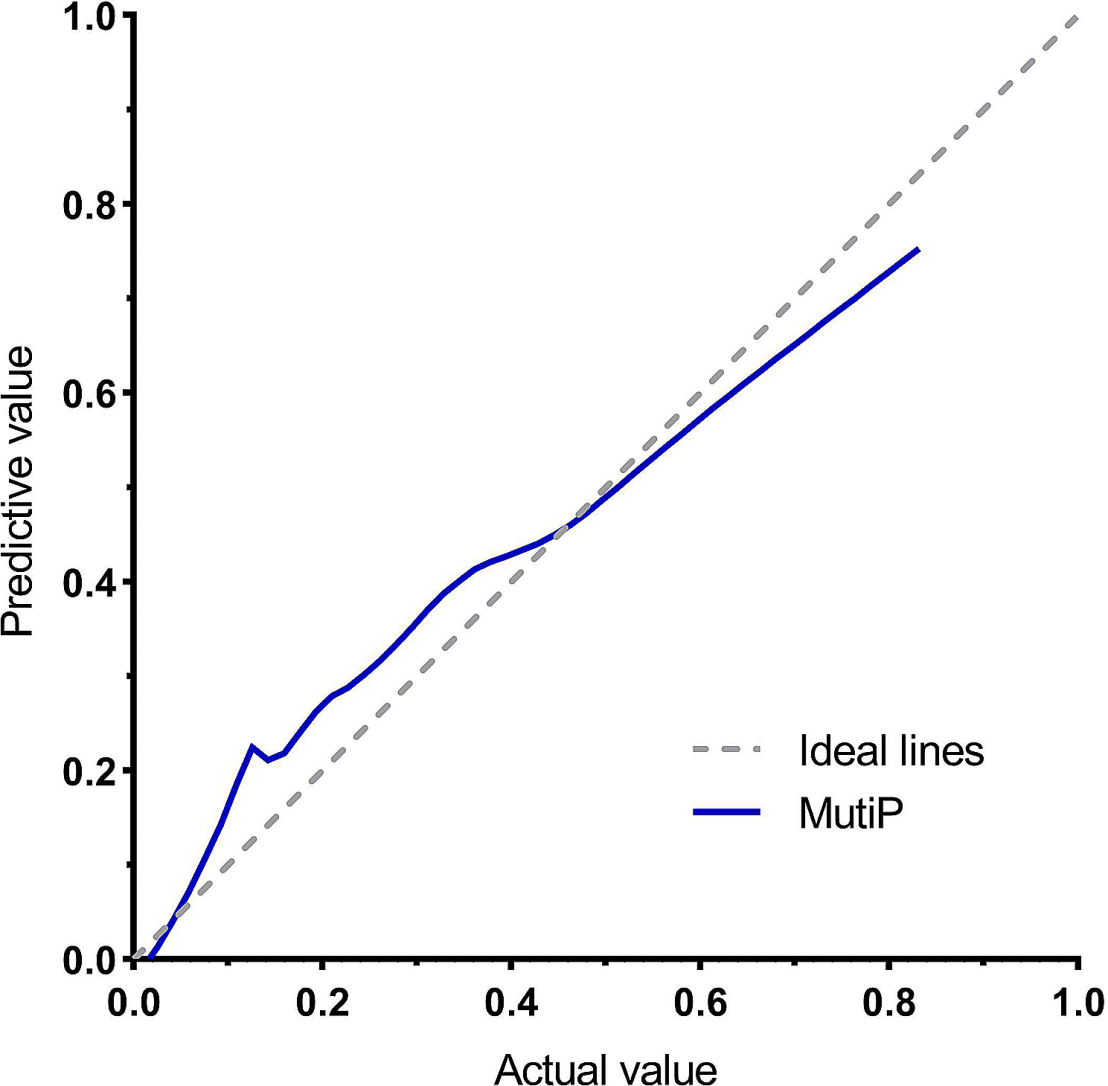
Calibration curve of the MultiP model. The MultiP curve (blue line) approaches the ideal lines, thus indicating a good calibration effect

**Fig. 10:**
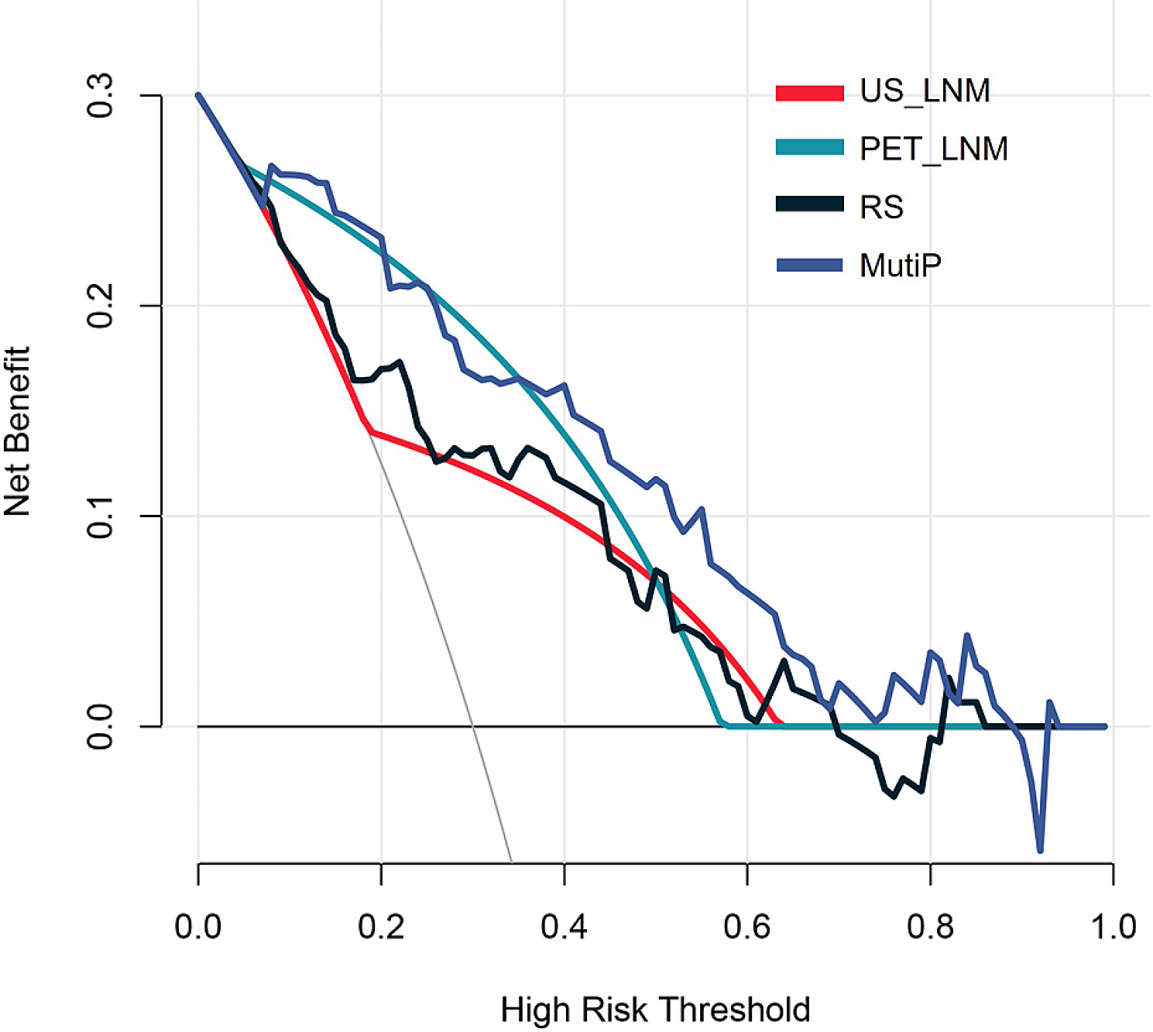
Decision curve analysis. The horizontal axis represents the range of decision thresholds, whereas the vertical axis indicates the benefit or loss associated with obtaining a positive result. The closer the curve is to the top and right boundaries, the better the performance of the model. Therefore, in this figure, the MultiP model outperforms US, PET, and RS.

ROC curve analysis of the nomogram of the parameters are shown in Table 3; Fig. 7. The MultiP nomogram had the highest AUC of 0.895 (95% CI = 0.840–0.951), surpassing all individual parameters, as shown in Fig. 8. The cut-off value with the maximum Youden index was 0.13, corresponding to a sensitivity of 96.15% and a specificity of 73.47%. The adjusted AUC by 1000 bootstrap resampling was 0.882. As shown in Fig. 9, the calibration curve indicated good calibration for the MultiP nomogram. Further decision curve analysis indicated that within the probability threshold range of 0.35–0.67, the MultiP nomogram provided greater net benefit for all patients, as shown in Fig. 10.

**Table 3:** AUC, cut-off value, sensitivity, and specificity of single- and MultiP nomograms

Parameters	AUC (95% CI)	*p*	Cut-off	Se (%)	Sp (%)
US_LNM	0.703 (0.614–0.782)	< 0.001	1	53.85	86.73
PET_LNM	0.814 (0.734–0.878)	< 0.001	1	92.31	70.41
RS	0.773 (0.689–0.843)	< 0.001	-1.12	61.54	87.76
MultiP	0.895 (0.840–0.951)	< 0.001	0.13	96.15	73.47

## Discussion

Breast cancer treatment includes surgery, radiotherapy, chemotherapy, adjuvant chemotherapy, and immunotherapy. The selection of breast cancer treatment plans is typically determined by various factors, including tumour staging, pathological type, and presence or absence of lymph node metastasis. Axillary lymph-node metastasis is a crucial indicator for breast cancer staging, and the number and status of axillary lymph-node metastasis are closely related to the prognosis of breast cancer patients [3]. Generally speaking, the greater the number of axillary lymph-node metastases, the worse the prognosis is for patients. Age may also influence the choice of treatment plans [15]. For instance, younger patients may be more inclined to choose local treatments such as breast-preserving surgery and radiotherapy, whereas older patients may prefer systemic treatments such as chemotherapy and endocrine therapy. To date, the primary focus has been on comprehensive treatment to improve prognosis or delay disease progression. Accurate assessment of axillary lymph-node metastatic burden in breast cancer is crucial for choosing appropriate comprehensive personalised treatment for patients. Currently, the development of a robust prediction model for axillary lymph-node metastasis is a focus of research both in China and worldwide.

A study [16] predicted early cervical squamous cell carcinoma by using ^18^F-FDG PET/CT radiomics, with an AUC of 0.91. There was also a report [17] using meta-analysis to summarise the predictive studies of ^18^F-FDG PET/CT radiomics for chest lymph-node metastasis in lung cancer, with an AUC of 0.94. Li et al. [6] reported the application of ^68^Ga-FAPI PET/CT in breast malignancies. Elboga et al. [18] compared the diagnostic accuracy of ^68^Ga-FAPI and ^18^F-FDG PET/CT in detecting breast cancer and found that ^68^Ga-FAPI performed better than ^18^F-FDG. Munter et al. [7] reported the clinical and economic effects of ^18^F-FES PET/CT for ER-positive breast cancer patients. Pedersen et al. [19] reported that ^18^F-FES was used for ER-positive breast cancer patients, thus resulting in higher lesion visibility. However, FAPI and FES are difficult to obtain, and the diagnosis and staging of breast cancer in clinical work still mainly rely on the application of ^18^F-FDG PET/CT.

Commonly used clinical imaging methods to assess axillary lymph-node metastatic burden in breast cancer are mostly based on the subjective experience of radiologists or low-dimensional semi-quantitative or quantitative analysis, with a large amount of deep and high-dimensional data not being fully utilised. Therefore, there is an urgent need for an accurate, preoperative, and non-invasive method that fully utilises high-dimensional data to predict axillary lymph-node metastatic burden in breast cancer, providing supplemental information for surgical decision-making, which aims to achieve precision diagnosis and treatment and to improve the quality of life for breast cancer patients.

The term “omics” originates in molecular biology and is used to describe the characteristics of DNA, RNA, proteins, and metabolites [8]. Radiomic features are products influenced by tissue genotype and phenotype, reflecting the biological characteristics of tumours [8]. In medical imaging research, radiomics involves deep mining of images to acquire clinically relevant data, providing potential imaging biomarkers for optimising diagnosis and treatment [20]. Compared to tissue-based biological markers, algorithm-based radiomic markers offer advantages such as non-invasiveness, real-time analysis, independence from and non-reliance on samples [21]. Recent studies have shown that radiomics demonstrated good predictive performance in assessing lymph-node metastasis in various cancers [22, 23].

Studies have indicated [9] the potential clinical value of radiomics in the diagnosis, staging, and treatment response assessment of breast cancer. Radiomics can improve the sensitivity of lymph-node metastasis diagnosis in breast cancer [24]. In earlier studies, our team has demonstrated that PET/CT-based radiomics in combination with US and clinical pathological features can predict axillary lymph-node metastasis in breast cancer [25]. However, PET/CT-based radiomics for axillary lymph-node metastatic burden in breast cancer has not been widely studied. Therefore, in the current study, we conducted a multivariate regression analysis of PET/CT-based radiomics, US, and clinical pathological features of breast cancer to establish a MultiP model for predicting axillary lymph-node metastatic burden.

One study [26] utilised US to predict axillary lymph-node burden in breast cancer, and the multivariate analysis demonstrated that having ≥ 3 abnormal lymph nodes was an independent influencing factor for HNB (OR = 18.385, 95% CI = 7.315–46.205, *p* < 0.05). Another study [27] utilised MRI to predict axillary lymph-node burden in breast cancer, and the multivariate analysis demonstrated that only vascular volume was an independent influencing factor for HNB (OR = 1.33, 95% CI = 1.03–1.67, *p* = 0.008). The findings of our study demonstrated a close relationship between positive axillary lymph nodes on US and axillary lymph-node metastatic burden. The US lymph-node positivity rate in the HNB group was significantly higher than that in the LNB group (χ^2^ = 19.867, *p* < 0.001), which was consistent with previous findings [28]. The PET lymph-node positivity rate in the HNB group was significantly higher than that in the LNB group (χ^2^ = 33.025, *p* < 0.001), which was consistent with previous findings [29].

Previous research has shown a stronger association between axillary lymph-node burden and imaging features [30]. In the current study, US lymph-node positivity, PET lymph-node positivity, and PET-based radiomics were all medical imaging features closely related to axillary lymph-node burden (*p* < 0.05), whereas other clinical pathological features showed no significant correlation, which was consistent with previous findings.

Our previous study [25] revealed a statistically significant difference in the PET-based RS predicting axillary lymph-node metastasis between the positive and negative groups. In this study, there was a statistically significant difference in the PET-based RS predicting axillary lymph-node burden between the HNB group and the LNB group (-1.04 ± 0.41 vs. -1.47 ± 0.41, t = -4.775, *p* < 0.001). The findings of this study indicated that US lymph-node positivity, PET lymph-node positivity, and RS were all independent influencing factors for HNB. The AUC of the MultiP model was 0.895, superior to those of US_LNM, PET_LNM, and RS models (AUC = 0.703, 0.814, 0.773, respectively), with statistically significant differences (Z = 2.888, 3.208, 3.804, respectively; *p* = 0.004, 0.002, < 0.001, respectively). Decision curve analysis indicated that the MultiP model provided higher net benefit for all patients.

It has been indicated [31] that the number of axillary lymph-node metastases is associated with the SUVmax of breast cancer nodules (*r* = 0.645, *p* < 0.001). However, in this study, there was no significant correlation between axillary lymph-node burden and SUV_max_. Another study suggested [32] an association between Ki67 and axillary lymph-node burden in breast cancer. However, in our study, the aforementioned factor showed no significant correlation with axillary lymph-node burden. A previous study [33] revealed that among different molecular subtypes of breast cancer, there was a higher likelihood of LNB in triple-negative breast cancer. However, the current study did not find a correlation between the molecular subtypes of breast cancer and the lymph-node burden possibly because of differences in patient inclusion/exclusion criteria.

This study had limitations. Firstly, it was a single-centre retrospective study, which could have introduced selection bias. Second, patients with bilateral, multifocal, and occult lesions were excluded. Third, owing to the limited amount of data, only internal validation was conducted, and expansion of data is necessary for further external validation.

## Conclusion

The multivariate model constructed based on ^18^F-FDG PET/CT-based radiomics in combination with US and clinical pathological features exhibited excellent predictive performance for axillary lymph-node metastatic burden in breast cancer and can serve as a reference tool for individualised precision treatment decision-making in clinical practice.

## Data Availability

The data that support the findings of this study are not openly available due to reasons of sensitivity and are available from the corresponding author upon reasonable request.
